# Socket Preservation after Tooth Extraction: Particulate Autologous Bone vs. Deproteinized Bovine Bone

**DOI:** 10.3390/bioengineering10040421

**Published:** 2023-03-27

**Authors:** Glauco Chisci, Arjeta Hatia, Elettra Chisci, Dafne Chisci, Paolo Gennaro, Guido Gabriele

**Affiliations:** 1 Centro Dentistico Chisci, Via Ricasoli 18, 58100 Grosseto, Italy; 2 Department of Orthodontics, University of Siena, 53100 Siena, Italy; 3 Department of Maxillofacial Surgery, University of Siena, 53100 Siena, Italy

**Keywords:** autologous bone, biomaterial, bundle bone, delayed implant, dental implant, deproteinized bone, graft, particulate bone, ridge preservation, socket preservation, tooth extraction

## Abstract

Background: The technique of socket preservation after tooth extraction allows for less volumetric decrease after tooth extraction. The aim of this retrospective study was to evaluate differences between alveolar socket preservation performed with deproteinized bovine bone graft and autologous particulate bone graft taken from the mandibular ramus. Materials and Methods: This retrospective study enrolled a total of 21 consecutive patients. A total of 11 patients underwent socket preservation with deproteinized bovine bone graft and collagen matrix (group A), and 10 patients underwent socket preservation performed with particulate autologous bone taken from the mandibular ramus and collagen matrix (group B). All patients received cone beam computed tomography (CBCT) before socket preservation and after four months. Alveolar bone width (ABW) values and alveolar bone height (ABH) values were measured at the first and second CBCT, and the reduction of the values in the two groups was compared. Statistical analysis was performed using Student’s *t*-test for independent variables, and *p* values < 0.05 were considered statistically significant. Results: There were no statistically significant differences between ABW reduction of group A and ABW reduction of group B (*t*-test value *p* = 0.28). There were no statistically significant differences between ABH reduction of group A and ABH reduction of group B (*t*-test value *p* = 0.10). Conclusions: In this retrospective study, no statistical differences were found between the group that received autologous particulate bone compared to the group that received deproteinized bovine bone in socket preservation.

## 1. Introduction

Today, implant dentistry represents the first choice in the treatment of edentulous patients. The treatment of edentulous patients with dental implants requires an appropriate preoperative evaluation in order to consider proper bone volume and adequate implant planning. In order to perform correct implant-supported prosthodontics, implant surgery should be correctly performed and should not be influenced by reduced bone volume. The extraction of teeth is usually followed by marked bone volume changes in the residual alveolar ridge, including severe volumetric changes in both height and thickness. Complicated extractions or extractions performed with the use of great force may lead to increased bone resorption after tooth extraction, and this aspect could lead to reduced bone volume. After tooth extraction, the height of the buccal alveolar ridge bone tends to decrease [1]. Further, the alveolar bone directly related to the periodontal tissue known as the bundle bone tends to disappear [2]. The entity and the development of the bundle bone have been studied and are reported in many papers. In their study, the authors Pietrokovski and Massler came to a similar conclusion. They emphasized that greater resorption and decrease in bone height occurred at the vestibular alveolar bone ridge of the molar region and less in the anterior frontal region after tooth extraction [3]. Schropp et al. described a decrease in the alveolar bone ridge up to 50% in width over 12 months of healing following dental extraction [4].

Many authors have evaluated the different stages of socket healing after tooth extraction in both animals and humans. Healing of the extraction socket is characterized by changes within the socket that lead to the formation of new bone tissue and changes outside the alveolar ridge that lead to a decrease in the width and height of the bone ridge [4,5]. These modifications of the hard tissues are reflected on the soft tissues as well, with impact on the aesthetics in cases of further implant-supported prosthodontics.

Regarding the internal phenomena and changes, a blood clot fills the socket immediately after extraction as a consequence of the rupture of the blood vessels of the periodontal ligament and of the apical foramen; consequently, the cells initiate a series of events that lead to the formation of a network of fibrin, which contributes with platelets to the formation of a stable clot in the first 24 h. Subsequent events in the course of healing include the formation of granulation tissue that will completely replace the blood clot in the following week and the formation of osteoid tissue at the base of the socket. With regard to the changes external to the alveolar socket, Araujo et al. observed that there is marked osteoclastic activity that results in resorption of the buccal and palatal alveolar bone ridges. Araujo and Lindhe in 2005 argued that, given that the buccal bone tissue of the alveolar socket after the extraction is made up of bundle bone, and this bone tissue is part of the periodontium, dental extraction renders this tissue useless, and resorption is a natural consequence [1].

On the other hand, other authors have argued that surgical trauma during extraction can determine the separation of the periosteum and its disconnection from the underlying bone surface. This can be the cause of vascular damage and acute inflammatory response, which in turn will lead to bone resorption. These stages have many characteristics in common with the formation of bone tissue following fractures of long bones [6]. A hard tissue bridge covers the marginal portion of the post-extraction site, and this phenomenon is well known as “corticalization” [7]. This phenomenon consists of a series of bone proliferative and resorption events that lead toward the formation of a lamina cortical bone. Numerous clinical and radiographic studies have described that these events lead to alterations in both height and width of the alveolar ridge [5,7]. The exact cause that leads to the realization of this phenomenon is still under discussion. A fundamental aspect not to be overlooked in reflections on this topic is the nature of the alveolar process, a tissue that has an intimate relationship dependent on dental elements and develops together with tooth eruption. Furthermore, as a consequence of the extraction of all permanent teeth, the alveolar process undergoes atrophy [8]. An aspect to consider is that the severity of bone resorption can represent an important problem for the clinician for various reasons. First, the absence of adequate levels of height of the residual alveolar ridge can lead to incorrect implant–prosthetic rehabilitation; second, aesthetics problems in the realization of implant-supported prosthetic rehabilitations can be caused by previous severe bone resorption events. This resorption process causes the bone crest to relocate to a more lingual position and influence the tridimensional aspect of bone volume [9,10].

Implant therapy can only be considered satisfactory when both functional and aesthetic objectives have been achieved: therefore, both adequate bone volume and favorable alveolar bone crest architecture are important considerations for obtaining prosthetic rehabilitation supported by implants both from the aesthetic and functional point of view.

For this reason, socket preservation procedures after tooth extraction have been designed and implemented to maintain the volume of the bone and gingival tissues, which can decrease following the extraction of the teeth. This procedure allows for the insertion of an implant fixture of greater diameter and length, compared to the post-extraction sockets that have not been preserved, and reduces the need for bone reconstruction simultaneous to implant insertion [10].

Over time, a great variety of techniques and biomaterials have been proposed to maintain the alveolar bone crest following the extraction of teeth. No statistically significant differences were noted between the various biomaterials, although collagen appears to be inadequate in neutralizing changes after tooth extraction [11]. The biomaterials used in socket preservation after tooth extraction maintain space and promote bone growth, primarily for their osteoconductive activity. Graft resorption and new bone formation can differ statistically significantly depending on the osteoconductive material [12,13,14]. Barone et al. published a randomized clinical trial that reported the ability of socket preservation in reducing the contours of the soft tissues after tooth extraction. The authors showed that despite the benefits obtained from this technique, a loss of width and height of the residual bone crest still occurs [15].

Barone et al. further compared the use of a full thickness flap with the use of a flapless technique in socket preservation and reported a greater resorption in terms of amplitude in the flap technique and a greater vertical resorption in the flapless technique [16]. Socket preservation of alveoli after tooth extraction allows for less volumetric decreases in bone ridge height and width [17,18]. This concept has been confirmed by numerous articles published in the literature that compare the preservation of alveoli after tooth extraction with biomaterial and spontaneous healing. For this reason, we conducted a single-center retrospective study on patients that received socket preservation after upper premolar extraction and delayed implant surgery, with the use of deproteinized bovine bone on one group and autologous bone on the other.

## 2. Materials and Methods

This is a retrospective study that involved patients that received extraction and socket preservation of the upper premolars and delayed implant surgery between 2020 and 2022. Patients presented fracture of the tooth as indication of treatment and agreed to tooth extraction, socket preservation and delayed dental implant and prosthodontics. Patients were asked to sign a written informed consent, in which treatment planning was discussed and benefit/risk ratio was explicated, with agreement to process personal data and images, and for publishing purposes, it was approved by the Institutional Review Board (scientific ethical committee of Centro Dentistico Chisci, Grosseto, Italy, 2020002; 17 September 2019). All study procedures complied with the principles stated in the Declaration of Helsinki “Ethical Principles for Medical Research Involving Human Subjects”, adopted by the 18th World Medical Assembly, Helsinki, Finland, June 1964, and as amended most recently by the 64th World Medical Assembly, Fortaleza, Brazil, October 2013. 

### 2.1. Inclusion Criteria 

Eligible patients (inclusion criteria) were selected among those older than 18 years, with absence of one mandibular third molar, systemically healthy, with a diagnosis of fractured upper premolar and indication of tooth extraction, socket preservation and delayed implant surgery with a preoperative documentation of CBCT, a postoperative CBCT and an indication of delayed dental implant. Patients were excluded if they: (i) required anticoagulation therapy; (ii) had systemic diseases that could interfere with oral tissue healing process/bleeding; (iii) were using bisphosfonates; (iv) were pregnant; (v) had mental/physical disabilities; (vi) had undergone radiation treatment to the head or neck region; (vii) showed infection of the interested tooth; (viii) had periodontitis; (ix) received antibiotic therapy in the last month. All of the patients were provided preoperative cone beam computed tomography (CBCT0) and received another CBCT four months after the extraction and socket preservation (CBCT1).

### 2.2. Tooth Extraction and Socket Preservation

None of the patients favored the habit of smoking, and no pathological health conditions were present. All the interventions were performed by the same surgeon (G.C.). Under local anesthesia with 1:100,000 articaine without the use of a flap, the tooth was gently extracted with luxation and pliers; the alveolar socket was smoothened and cleansed with irrigation of 0.9 NaCl for 30 s.

**Figure 1:**
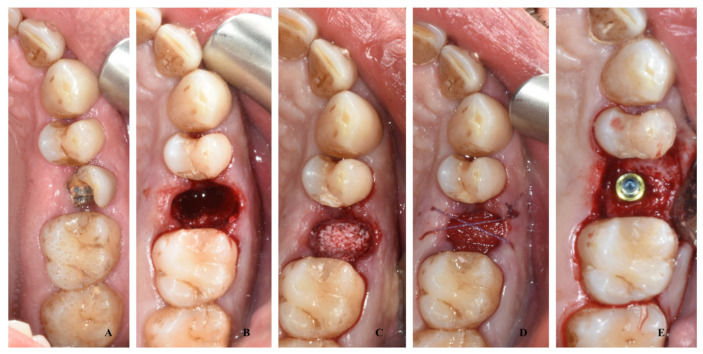
A case of socket preservation performed with a sponge of deproteinized bovine bone inside the alveolar socket covered with a resorbable collagen matrix and the insertion of a dental implant after four months: preoperative image (**A**); image after the extraction (**B**); image after the filling with deproteinized bovine bone (**C**); image after placement of collagen matrix and suture (**D**); image after recovery at time of implant surgery (**E**).

A total of 11 patients (group A) after tooth extraction received deproteinized bovine bone (Bio-oss, Geistlich Pharma Italy, Thiene, Italy) inside the alveolar socket covered with a resorbable collagen matrix (hemocollagene, septodont, Mataro, Spain) with a diameter of 8 mm sutured at the soft tissue with resorbable stitches (Figure 1).

**Figure 2:**
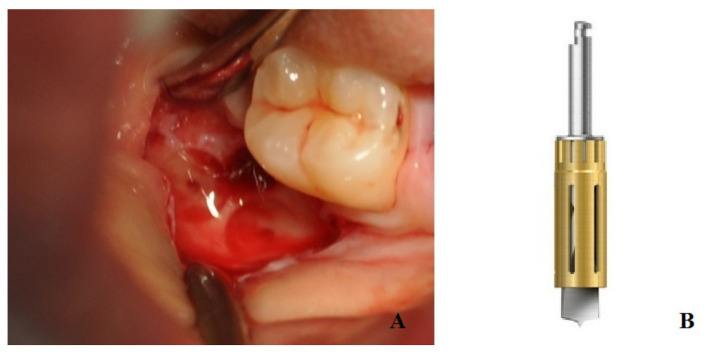
The figure shows bone harvest from the mandibular ramus performed with bur mounted on a handpiece (**A**); detail of the bur used for this procedure (**B**).

**Figure 3:**
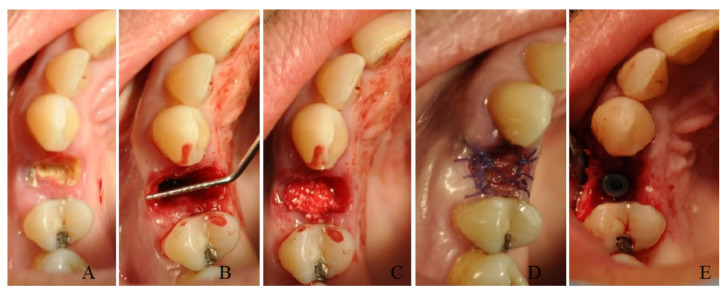
A case of socket preservation performed with autologous bone harvested from the mandible placed inside the alveolar socket covered with a resorbable collagen matrix and the insertion of the dental implant after four months: preoperative image with decayed root (**A**); image after root extraction (**B**); image of socket filled with autologous bone graft (**C**); suture and collagen matrix (**D**); image at the time of implant surgery, with detail of cap screw (**E**).

A total of ten patients (group B) after tooth extraction received bone harvesting from the mandibular ramus on the side of the extraction: under local anesthesia with 1:100,000 articaine, a small flap was elevated with a bur mounted on a handpiece (Autobone collector, Osstem, Micerium, Avegno, Italy), and an autologous particulated cortical bone graft was harvested (Figure 2). This bone graft was then placed inside the alveolar socket covered with a resorbable collagen matrix diameter of 8 mm sutured at the soft tissue with resorbable stitches (Figure 3).

Sutures were removed at 14 days after surgery. Patients received ibuprofen 600 mg for treatment of postoperative pain and swelling both at the extraction and socket preservation and at the time of implant surgery. 

### 2.3. Data Collection

**Figure 4:**
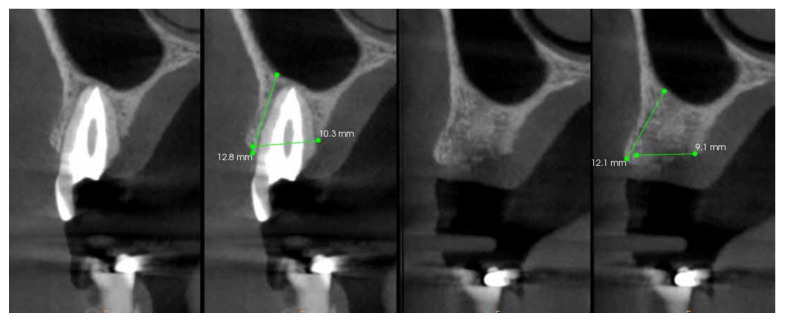
Measurements performed on the CBCT0 and CBCT1: alveolar bone width (ABW) and alveolar bone height (ABH).

Each patient received two measurements in millimeters on CBCT0 and CBCT1: alveolar bone width (ABW), measured as distance between the most coronal point on the vestibular cortical bone and the most coronal point on the palatal cortical bone; and alveolar bone height (ABH), measured as the most coronal point on the vestibular cortical bone and the cortical bone of the maxillary sinus (Figure 4). The alveolar bone width reduction (ABWR) after four months was measured as a subtraction between ABW measured on CBCT0 and ABW measured on CBCT1. The alveolar bone height reduction (ABHR) after four months was measured as a subtraction between ABH measured on CBCT0 and ABH measured on CBCT1.

### 2.4. Statistical Analysis

The statistical analysis was performed using Student’s *t*-test for independent variables, and *p* values < 0.05 were considered statistically significant. The software MedCalc version 9.5.2.0 (MedCalcSoftware, Mariakerke, Belgium) was used for statical analysis. 

## 3. Results

In the present study, 21 teeth were extracted in 21 patients, and socket preservations were performed on all patients. No major complications were reported after socket preservation, and all wounds appeared healthy at suture removal. All patients received delayed implant surgery after four months. Each patient provided preoperative CBCT, and another CBCT was performed four months after extraction and socket preservation in order to perform implant surgery.

Of these patients, 11 patients received socket preservation of alveoli performed with deproteinized bovine bone, and 10 patients received socket preservation of alveoli performed with particulate autologous bone harvested from the mandibular ramus.

**Figure 5:**
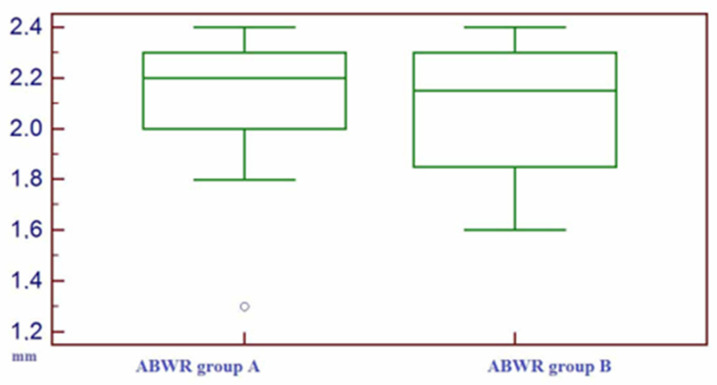
Box–whisker plot representing the distribution of alveolar bone width reduction (ABWR) on group A and on group B.

The results of ABWR were (mean value +/− standard deviation) 2.13 +/− 0.25 mm (range 1.3–2.5 mm) for group A and (mean value +/- standard deviation) 2.08 +/− 0.27 mm (range 1.6–2.4 mm) for group B. There were no statistically significant differences between the ABWR of group A and ABWR of group B (*t*-test value *p* = 0.28) (Figure 5).

**Figure 6:**
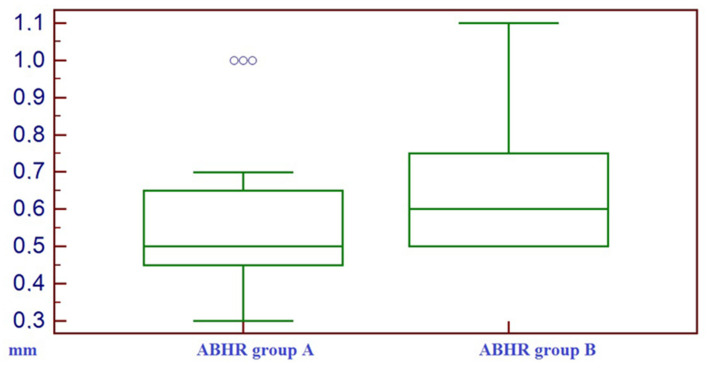
Box–whisker plot representing the distribution of alveolar bone height reduction (ABHR) on group A and on group B.

The results of ABHR were (mean value +/− standard deviation) 0.59 +/− 0.22 mm for group A and (mean value +/− standard deviation) 0.70 +/− 0.23 mm for group B. There were no statistically significant differences between the ABHR of group A and the ABHR of group B (*t*-test value *p* = 0.10) (Figure 6).

In all the cases reported, implant surgery was performed four months after tooth extraction and socket preservation. In all cases, no implant dehiscence was noted, and no need for bone augmentation was required.

## 4. Discussion

Healing at an extraction site is characterized by the new organization, proliferation and maturation of the oral tissues, resulting in tridimensional changes to the alveolar bone and gingival tissues [19,20]. The amount of horizontal and vertical alveolar bone change is directly interlinked, as vertical crestal resorption can occur as a direct result of damage to the extraction socket or due to a complex pattern of osteoclastic remodeling activity on either the inner or outer socket wall, leading to both vertical and horizontal dimensional changes [9].

Socket preservation is a technique defined as alveolar ridge preservation within the bone envelope remaining after tooth extraction with the purpose of reducing bone resorption in order to perform a correct implant-supported prosthesis [21]. The need of socket preservation immediately after tooth extraction should be determined by the aesthetic, functional and risk-related viewpoint [13,21]: in the case of a treatment plan with implant-supported prosthodontics of the extracted tooth with risk of excessive resorption after tooth extraction and/or aesthetic impact of the tooth, socket preservation is suggested [22]. Alenazi et al. underlined the need for socket preservation after tooth extraction in the case of future implant prosthesis in order to have correct bone volume [23].

Once the need of socket preservation for future implant surgery for tooth replacement is determined, the choice of biomaterial and technique to be used should be discussed. Majzoub et al. evaluated many different graft biomaterials to be used in socket preservation after tooth extraction, with better results for all graft materials compared to spontaneous healing [24]. Spontaneous recovery has been commonly used as a control to evaluate performance of the graft material in socket preservation, with better results in the test site compared to the spontaneous-healing control site. 

This technique appeared to be effective even for the teeth affected by severe periodontitis, with results of an adequate level of keratinized soft tissues [25]. Periodontitis represents a great contraindication for implant surgery in oral and maxillofacial surgery. This pathology is related to many postoperative complications and reduced success rate [26,27]. Periodontal patients should undergo classification and periodontitis treatment, and after recovery and positive follow-up, the patients may be eligible for implant prosthodontics.

A wide variety of techniques and biomaterials have been proposed over time to maintain the alveolar bone ridge following tooth extraction; however, the lack of superiority of any particular technique or biomaterial among the others has led to many existing procedures with the same purpose and different methodologies underlining the advantages of any one technique compared to the others. In a recent study, Covani et al. concluded that the collagen plug within an intact alveolus might be sufficient in preventing extensive tridimensional collapse of the alveolar bone [28]. In order to reduce the use of a xenograft and support the use of autologous grafts, recently, the use of platelet-rich fibrin has been introduced in socket preservation after tooth extraction [29]. Platelet-rich fibrin is a natural endearing autologous composite material that accelerates all the physiological healing phenomena and requires the execution of a preparatory technique of the patient’s blood. However, criticism has been raised regarding the possible benefits this technique can confer compared to other socket preservation techniques and other biomaterials [29]. The use of platelet-rich fibrin however has shown some small benefits compared with spontaneous healing after tooth extraction [29].

Another important matter of discussion of the regenerative techniques in dentistry is the concept of space provision in the surgical site. An adequate scaffold that supports the regenerative process is suggested to be of use in socket preservation, as it emphasizes the importance of the type of carrier in the three-dimensional distribution of particles and space provision in new bone formation, especially during the early stages [30].

The use of autologous bone in oral and maxillofacial surgery is not a novelty [31]. Bone blocks or particulate bone have been commonly used for bone augmentation. The use of particulate autologous bone in socket preservation after tooth extraction is not so common, albeit much research on socket preservation has been reported in the last years. The main limits of using particulate bone from the patient consist of its greater ease of resorption, the morbidity of the harvesting technique (the opening of a second surgical site) and the small amount of tissue that can be harvested from intraoral sites. Further, some patients negate the use of different biomaterials for regenerative purposes, and the lack of patient consent to the use of biomaterials has opened the field of using autologous bone. Further, with the spread of the use of autologous platelet-rich fibrin, the harvesting of autologous bone from the mandibular ramus does not appear to be an invasive method.

In this study, we found similar values of ABWR and ABHR in both groups, meaning that a possible use of particulate autologous bone in socket preservation could lead to results similar to deproteinized bovine bone. The use of a collagen matrix in the coronal site as a “seal” of the intervention could play a role. the seal from oral saliva and food impaction could lead to an advantaged recovery, compared with spontaneous healing, and could jeopardize the differences from particulated autologous bone and deproteinized bovine bone in the limited intervention of socket preservation [32]. This concept was already suggested by Covani et al. regarding the limits of an intact alveolus after tooth extraction [28]. 

The results of socket preservation presented in this paper are in line with the results from the international literature. 

Falacho et al., in their animal study, reported significant difference among four different biomaterials used in femoral socket preservation, with a superiority of mp3, Gen-Os and Apatos compared to the rest in terms of new bone formation, suggesting a promising regenerative effect of collagenated porcine heterologous bone grafts [33]. In our study, we did not perform histomorphometric evaluation of the operated socket, but we measured the bone volume before and after the socket preservation in patients, and we confirmed the benefit of this technique for implant purposes.

The strength of this paper is the routine use of autologous particulate bone harvested with a simple drill from the mandibular ramus (Figure 2) and the use of CBCT referral for each measurement reported. Although many papers have reported the possible benefits of socket preservation after tooth extraction, few papers have used standardized preoperative and postoperative CBCT images to evaluate the measurements [34]. The majority of socket preservation research has compared preoperative measurements performed on intraoral bidimensional radiography with postoperative bidimensional or three-dimensional images, or on a resin template with periodontal probe measurements and clinical measurements of the bone width [16]. The advantage of the procedures exposed in this study is the harvesting of bone tissue from an intraoral site to perform socket preservation after tooth extraction. Regarding the amount of harvestable tissue, in all cases in the inclusion criteria, the absence of a mandibular third molar played a role in order in that there were no limits for harvesting from the mandibular ramus. MacBeth et al., in their randomized, single-blind controlled clinical trial, used CBCT immediately after tooth removal and four months after extraction. This methodology was revealed to be more accurate as the absence of a tooth in the images allowed for more detailed measurements [34].

One limitation of this study included the small groups of subjects; however, many studies regarding socket preservation techniques are conducted in vivo or in animal research. Harvesting of the mandibular ramus was another possible limitation. 

Although harvesting from mandibular ramus allows for an adequate volume of bone to be harvested for socket preservation after tooth extraction, it has numerous flaws, such as the presence of a third molar, possible negation from patients, or the presence of a previously harvested site. Future randomized and prospective studies with larger, multicentered patients are needed to test the validity of this procedure. In this limited study, buccolingual width and bone crest height were measured on CBCT by one operator. This procedure has a limitation in that it is potentially fallacious in error, due to the retrieval of referral points on the image. Furthermore, in this paper, many inclusion criteria were introduced in order to produce a correct harvest bone volume from the mandibular ramus with the exception of patients with the presence of a third molar. However, autologous bone harvesting remains an attractive source of material for post-extractive socket preservation, bone augmentation procedures and bone regeneration techniques. The results of this paper suggest comparable results to deproteinized bovine bone and autologous particulate bone in socket preservation after tooth extraction. This could underline the role of a collagen seal in the coronal site of the alveolus, regardless of the material used to fill the socket, but further studies are required to assess this theory.

## 5. Conclusions

In this study, results of socket preservation after tooth extraction performed with biomaterial versus socket preservation performed with autologous bone harvested from the mandibular ramus with a drill were reported. CBCT referrals reported no statistical differences between the two techniques, with a possible role of the collagen bone matrix. The characteristics between the two techniques as well as advantages and disadvantages are for the clinician to consider in order to choose the most appropriate technique to reduce morbidity for the patient and to achieve the most desired bone volume available for implant surgery.

## Data Availability

Not applicable.

## References

[1] Araujo M.G., Lindhe J. (2005). Dimensional ridge alterations following tooth extraction. An experimental study in the dog. J. Clin. Periodontol..

[2] Cardaropoli G., Wennström J.L., Lekholm U. (2003). Periim-plant bone alterations in relation to inter-unit distances. Clin. Oral Implant. Res..

[3] Pietrokovski J., Massler M. (1967). Alveolar ridge resorption following tooth extraction. J. Prosthet. Dent..

[4] Schropp L., Wenzel A., Kostopoulos L., Karring T. (2003). Bone healing and soft tissue contour changes following single-tooth extraction: A clinical and radiographic 12-month prospective study. Int. J. Periodontics Restor. Dent..

[5] Johnson K. (1969). A study of the dimensional changes occurring in the maxilla following tooth extraction. Aust. Dent. J..

[6] Hollinger J., Wong M.E. (1996). The integrated processes of hard tissue regeneration with special emphasis on fracture healing. Oral Surg. Oral Med. Oral Pathol. Oral Radiol. Endodontol..

[7] Ohnishi H., Fujii N., Futami T., Taguchi N., Kusakari H., Maeda T. (2000). A Histochemical Investigation of the Bone Formation Process by Guided Bone Regeneration in Rat Jaws. Effect of PTFE Membrane Application Periods on Newly Formed Bone. J. Periodontol..

[8] Tallgren A. (1972). The continuing reduction of the residual alveolar ridges in complete denture wearers: A mixed-longitudinal study covering 25 years. J. Prosthet. Dent..

[9] Cardaropoli G., Araújo M., Lindhe J. (2003). Dynamics of bone tissue formation in tooth extraction sites. An experimental study in dogs. J. Clin. Periodontol..

[10] Covani U., Ricci M., Bozzolo G., Mangano F., Zini A., Barone A. (2011). Analysis of the pattern of the alveolar ridge remodelling following single tooth extraction. Clin. Oral Implant. Res..

[11] Vignoletti F., Matesanz P., Rodrigo D., Figuero E., Martin C., Sanz M. (2012). Surgical protocols for ridge preservation after tooth extraction. A systematic review. Clin. Oral Implant. Res..

[12] Barone A., Aldini N.N., Fini M., Giardino R., Guirado J.L.C., Covani U. (2008). Xenograft versus Extraction Alone for Ridge Preservation after Tooth Removal: A Clinical and Histomorphometric Study. J. Periodontol..

[13] Chisci G., Fredianelli L. (2022). Therapeutic Efficacy of Bromelain in Alveolar Ridge Preservation. Antibiotics.

[14] Lindgren C., Sennerby L., Mordenfeld A., Hallman M. (2009). Clinical histology of microimplants placed in two different biomaterials. Int. J. Oral Maxillofac. Implant..

[15] Barone A., Ricci M., Tonelli P., Santini S., Covani U. (2013). Tissue changes of extraction sockets in humans: A comparison of spontaneous healing vs. ridge preservation with secondary soft tissue healing. Clin. Oral Implant. Res..

[16] Barone A., Toti P., Piattelli A., Iezzi G., Derchi G., Covani U. (2014). Extraction Socket Healing in Humans After Ridge Preservation Techniques: Comparison Between Flapless and Flapped Procedures in a Randomized Clinical Trial. J. Periodontol..

[17] Barone A., Toti P., Quaranta A., Alfonsi F., Cucchi A., Negri B., Di Felice R., Marchionni S., Guirado J.L.C., Covani U. (2017). Clinical and Histological changes after ridge preservation with two xenografts: Preliminary results from a multicentre randomized controlled clinical trial. J. Clin. Periodontol..

[18] Barone A., Toti P., Quaranta A., Alfonsi F., Cucchi A., Calvo-Guirado J.L., Negri B., Di Felice R., Covani U. (2016). Volumetric analysis of remodelling pattern after ridge preservation comparing use of two types of xenografts. A multicentre randomized clinical trial. Clin. Oral Implant. Res..

[19] Avila-Ortiz G., Chambrone L., Vignoletti F. (2019). Effect of alveolar ridge preservation interventions following tooth extraction: A systematic review and meta-analysis. J. Clin. Periodontol..

[20] Avila-Ortiz G., Gonzalez-Martin O., Dds E.C., Wang H. (2020). The peri-implant phenotype. J. Periodontol..

[21] Juodzbalys G., Stumbras A., Goyushov S., Duruel O., Tözüm T.F. (2019). Morphological Classification of Extraction Sockets and Clinical Decision Tree for Socket Preservation/Augmentation after Tooth Extraction: A Systematic Review. J. Oral Maxillofac. Res..

[22] Santhanakrishnan M., Ramesh N., Kamaleeshwari R., Subramanian V. (2021). Variations in Soft and Hard Tissues following Immediate Implant Placement versus Delayed Implant Placement following Socket Preservation in the Maxillary Esthetic Region: A Randomized Controlled Clinical Trial. BioMed Res. Int..

[23] Alenazi A., Alotaibi A.A., Aljaeidi Y., Alqhtani N.R. (2022). The need for socket preservation: A systematic review. J. Med. Life.

[24] Majzoub J., Ravidà A., Starch-Jensen T., Tattan M., Del Amo F.S.-L. (2019). The Influence of Different Grafting Materials on Alveolar Ridge Preservation: A Systematic Review. J. Oral Maxillofac. Res..

[25] Wei Y., Xu T., Hu W., Zhao L., Wang C., Chung K.-H. (2021). Socket Preservation Following Extraction of Molars with Severe Periodontitis. Int. J. Periodontics Restor. Dent..

[26] Chisci G., Gabriele G., Gennaro P. (2023). Periodontal disease before and after fractures of the mandible. Br. J. Oral Maxillofac. Surg..

[27] Papapanou P.N., Sanz M., Buduneli N., Dietrich T., Feres M., Fine D.H., Flemmig T.F., Garcia R., Giannobile W.V., Graziani F. (2018). Periodontitis: Consensus report of workgroup 2 of the 2017 World Workshop on the Classification of Periodontal and Peri-Implant Diseases and Conditions. J. Periodontol..

[28] Covani U., Giammarinaro E., Panetta D., Salvadori P.A., Cosola S., Marconcini S. (2022). Alveolar Bone Remodeling with or without Collagen Filling of the Extraction Socket: A High-Resolution X-ray Tomography Animal Study. J. Clin. Med..

[29] Alrayyes Y., Al-Jasser R. (2022). Regenerative Potential of Platelet Rich Fibrin (PRF) in Socket Preservation in Comparison with Conventional Treatment Modalities: A Systematic Review and Meta-Analysis. Tissue Eng. Regen. Med..

[30] Palma P.J., Matos S.M., Ramos C.J., Figueiredo M.H., Krauser J., Guerra F.A.D.R.A. (2010). New formulations for space provision and bone regeneration. Biodental. Eng..

[31] Bhandi S., Patil S., Jafer M.A., Salem R.M., Hakami F.B., Ageeli R.E., Alhazmi T.A. (2022). Techniques for Extraction Socket Regeneration for Alveolar Ridge Preservation. J. Contemp. Dent. Pract..

[32] Sassano P., Gennaro P., Chisci G., Gabriele G., Aboh I.V., Mitro V., di Curzio P. (2014). Calvarial Onlay Graft and Submental Incision in Treatment of Atrophic Edentulous Mandibles. J. Craniofacial Surg..

[33] Falacho R., Palma P., Marques J., Figueiredo M., Caramelo F., Dias I., Viegas C., Guerra F. (2021). Collagenated Porcine Heterologous Bone Grafts: Histomorphometric Evaluation of Bone Formation Using Different Physical Forms in a Rabbit Cancellous Bone Model. Molecules.

[34] MacBeth N.D., Donos N., Mardas N. (2022). Alveolar ridge preservation with guided bone regeneration or socket seal technique. A randomised, single-blind controlled clinical trial. Clin. Oral Implant. Res..

